# Supplementary material for: Interventions with Music in PECTus excavatum treatment (IMPECT trial): a study protocol for a randomised controlled trial investigating the clinical effects of perioperative music interventions
Source: BMJ Open. 2020 Jul 8;10(7):e036380. doi: 10.1136/bmjopen-2019-036380 (PMC7348468; doi:10.1136/bmjopen-2019-036380)
Supplement: Author's manuscript [file bmjopen-2019-036380.draft_revisions.pdf]

BMJ Open is committed to open peer review. As part of this commitment we make the peer review history of every article we publish publicly available.

When an article is published we post the peer reviewers' comments and the authors' responses online. We also post the versions of the paper that were used during peer review. These are the versions that the peer review comments apply to.

The versions of the paper that follow are the versions that were submitted during the peer review process. They are not the versions of record or the final published versions. They should not be cited or distributed as the published version of this manuscript.

BMJ Open is an open access journal and the full, final, typeset and author-corrected version of record of the manuscript is available on our site with no access controls, subscription charges or pay-per-view fees (http://bmjopen.bmj.com).

If you have any questions on BMJ Open's open peer review process please email info.bmjopen@bmj.com

BMJ Open

# Interventions with Music in Pectus Excavatum Treatment (IMPECT trial): A study protocol for a randomized controlled trial investigating the clinical effects of perioperative music interventions


| | |
|---|---|
| Journal: | *BMJ Open* |
| Manuscript ID | bmjopen-2019-036380 |
| Article Type: | Protocol |
| Date Submitted by the Author: | 13-Dec-2019 |


| | |
|---|---|
| Complete List of Authors: | Billar, Ryan; Erasmus MC Sophia Children Hospital, Pediatric Surgery<br>Kühlmann, Rosalie; Saint Antonius Hospital, Anaesthesiology<br>Schnater, Marco; Erasmus MC Sophia Children Hospital, Paediatric Surgery<br>Vlot, John; Erasmus MC Sophia Children Hospital, Paediatric Surgery<br>Tomas, Jeremy; Erasmus MC Sophia Children Hospital, Anaesthesiology<br>Zijp, Gerda; Haga Hospital Juliana Childrens Hospital, Paediatric Surgery<br>Rad, Mandana; Haga Hospital Juliana Childrens Hospital, Anaesthesiology<br>de Beer, Sjoerd; Emma Childrens Hospital AMC, Paediatric Surgery<br>Stevens, Markus; Emma Childrens Hospital AMC, Anaesthesiology<br>Poley, Marten; Erasmus University Rotterdam, Institute for Medical Technology Assessment; Erasmus MC Sophia Children Hospital,<br>van Rosmalen, Joost; Erasmus MC, Biostatistics<br>Jeekel, Johannes; Erasmus MC,<br>Wijnen, Rene; Erasmus MC Sophia Children Hospital, Paediatric Surgery |


| | |
|---|---|
| Keywords: | Paediatric thoracic surgery < PAEDIATRIC SURGERY, Paediatric anaesthesia < PAEDIATRICS, PAIN MANAGEMENT |
| | |


SCHOLARONE™
Manuscripts

*I, the Submitting Author has the right to grant and does grant on behalf of all authors of the Work (as defined in the below author licence), an exclusive licence and/or a non-exclusive licence for contributions from authors who are: i) UK Crown employees; ii) where BMJ has agreed a CC-BY licence shall apply, and/or iii) in accordance with the terms applicable for US Federal Government officers or employees acting as part of their official duties; on a worldwide, perpetual, irrevocable, royalty-free basis to BMJ Publishing Group Ltd ("BMJ") its licensees and where the relevant Journal is co-owned by BMJ to the co-owners of the Journal, to publish the Work in this journal and any other BMJ products and to exploit all rights, as set out in our licence.*

*The Submitting Author accepts and understands that any supply made under these terms is made by BMJ to the Submitting Author unless you are acting as an employee on behalf of your employer or a postgraduate student of an affiliated institution which is paying any applicable article publishing charge ("APC") for Open Access articles. Where the Submitting Author wishes to make the Work available on an Open Access basis (and intends to pay the relevant APC), the terms of reuse of such Open Access shall be governed by a Creative Commons licence – details of these licences and which Creative Commons licence will apply to this Work are set out in our licence referred to above.*

*Other than as permitted in any relevant BMJ Author's Self Archiving Policies, I confirm this Work has not been accepted for publication elsewhere, is not being considered for publication elsewhere and does not duplicate material already published. I confirm all authors consent to publication of this Work and authorise the granting of this licence.*

For peer review only

# Interventions with Music in Pectus Excavatum Treatment (IMPECT trial): A study protocol for a randomized controlled trial investigating the clinical effects of perioperative music interventions

RJ Billar[1], AYR. Kühlmann[1,10], JM Schnater[1], J Vlot[1], JJP Tomas[2], GW Zijp[6], M Rad[7], SA de Beer[8], MF Stevens[9], MJ Poley[1,5], J van Rosmalen[3], JF Jeekel[4], RMH Wijnen[1]

**Author affiliations**

Erasmus University Medical Centre – Sophia Children's Hospital, Rotterdam, the Netherlands:

1 department of Paediatric Surgery; 2 department of Anaesthesiology

Erasmus University Medical Centre, Rotterdam, the Netherlands:

3 department of Biostatistics; 4 department of Neuroscience

Erasmus University, Rotterdam, the Netherlands:

5 institute for Medical Technology Assessment

Haga Hospital – Juliana Children's Hospital, the Hague, the Netherlands:

6 department of Surgery; 7 department of Anaesthesiology

Academic Medical Centre - Emma Children's Hospital, Amsterdam, the Netherlands:

8 department of Surgery; 9 department of Anaesthesiology

St Antonius Hospital, Nieuwegein, The Netherlands:

10 department of Anaesthesiology

Corresponding author: RJ Billar

Department of paediatric surgery

Erasmus University Medical Centre-Sophia Children's Hospital

Doctor Molewaterplein 40, 3015GD Rotterdam, the Netherlands

Phone: 00310-7036783

Fax: 00310-7036802

Email: r.billar@erasmusmc.nl



For peer review only - http://bmjopen.bmj.com/site/about/guidelines.xhtml

## Abstract

**Introduction:** Pectus excavatum repair is associated with substantial postoperative pain, despite the use of epidural analgesia and other analgesic regimens. Perioperative recorded music interventions have been shown to alleviate pain and anxiety in adults, but evidence for children and adolescents is still lacking. This study protocol describes a randomized controlled trial that evaluates effects of recorded music interventions on postoperative pain relief in in children and adolescents after pectus excavatum repair.

**Methods:**

A multicentre, randomized controlled trial was set up comparing the effects of perioperative recorded music interventions in addition to standard care with those of standard care only in patients undergoing a Nuss-procedure for pectus excavatum repair. One hundred and seventy subjects (12-18 years of age) will be included in three centres in the Netherlands. Patient inclusion has started in November 2018, and is ongoing. The primary outcome is self-reported perceived pain measured on the Visual Analogue Scale. Secondary outcomes are anxiety level, analgesics consumption, vital parameters such as heart rate, blood pressure and respiratory rate, length of hospital stay, postoperative complications, quality of life, and cost-effectiveness.

**Ethics and dissemination**

This study is being conducted in accordance with the Declaration of Helsinki. The medical ethics review board of Erasmus University Medical Centre Rotterdam, the Netherlands, has approved this protocol. Results will be disseminated via peer-reviewed scientific journals and conference presentations.

**Trial registration number** NL6863


For peer review only

**Strengths and limitations**

- The IMPECT trial is the first multicentre randomized controlled trial evaluating the effects of recorded music interventions on pain experience in older children before, during and after pectus excavatum repair with the Nuss-procedure.
- Data will be collected during hospitalization and up until 3 months postoperatively to shed light on the effect of perioperative recorded music interventions during hospitalization and after hospitalization evaluating both short-term and potentially long-term effects.
- The study participants and participating surgeons are not blinded to the interventions, which is a limitation; however, the anaesthesiologists and pain specialists will be blinded to the study arm allocation.

## *Introduction*

Pectus excavatum (PE) is the most common congenital chest wall deformity affecting 0.1-0.8% of live births, affecting boys more than girls. Operative repair is indicated when symptoms or signs of heart and/or lung dysfunction are present,(1) or when the patient is much concerned about the cosmetic appearance and psychosocial problems occur.(2, 3) The optimal age for repair is between 12 and 16 years.(4) Numerous surgical techniques have been developed to correct PE, of which the Nuss-procedure is now among the most commonly employed techniques. (5, 6) The Nuss-procedure involves inserting a convex steel bar beneath the sternum, to reposition the sternum anteriorly and thereby effectively correcting the deformity.(7) It is associated, however, with substantial postoperative pain, despite the use of epidural analgesia or patient-controlled intravenous opioid administration.(8, 9) Pain management is the critical component of postoperative care as postoperative pain has implications for activity and quality of life (10) and is the primary factor determining the length of hospital stay.(11) Therefore, interest is growing in finding new ways to alleviate postoperative pain, such as perioperative music interventions. In previous studies in adult surgical patients, recorded music interventions reduce pain medication consumption and improve the management of pain and anxiety.(12-20) However in children and adolescents undergoing surgery, a definite conclusion about the effect of recorded music interventions has yet to be drawn.(21) Especially in paediatric surgical procedures associated with substantial postoperative pain, such as the Nuss-procedure, music



For peer review only

interventions might be effective in reducing children's pain and anxiety. We designed a multicentre, randomized controlled trial (IMPECT trial) to evaluate whether adjuvant recorded music interventions are indeed associated with less postoperative pain in children and adolescents undergoing the Nuss-procedure for PE repair.

## *Methods and analysis*

### Study design

The IMPECT trial is a randomized controlled trial with two study arms, designed to compare the effects on postoperative pain of perioperative recorded music interventions in addition to standard care (intervention group) versus standard care (control group) – prior, during and after the Nuss-procedure for PE repair. We will include one hundred and seventy subjects children and adolescents (12-18 year of age) operated on in three centres in the Netherlands: the Erasmus University Medical Centre-Sophia Children's Hospital, Rotterdam; Haga Hospital-Juliana Children's Hospital, The Hague; Academic Medical Centre-Emma Children's Hospital, Amsterdam. Patient inclusion has started in November 2018, and is ongoing.

This study protocol follows the Standard Protocol Items: Recommendations for Interventional Trials (SPIRIT) guidelines (see SPIRIT checklist in online supplemental files). The underlying protocol follows the Consolidated Standards of Reporting Trials (CONSORT) guidelines for non-pharmacological treatments. This trial was registered on trialregister.nl (NL6863).

### Randomization, blinding and treatment allocation

A parallel randomization with equal allocation ratio is being carried out to individually allocate subjects to either the intervention or the control group. An online web-based randomization program (ALEA; FormVision, Abcoude, the Netherlands) generates the random allocation sequence by the use of random block size randomization and is stratified by centre with an equal allocation-ratio per centre in both study arms. Allocation concealment will be ensured, as the service will not release the randomization code until the patient has been recruited into the trial. The anaesthesiologists and pain specialists involved do not have access to the randomization program and are blinded to the subject's study arm allocation, as well as the person analysing the data.



For peer review only - http://bmjopen.bmj.com/site/about/guidelines.xhtml

**Interventions**

Subjects in the intervention arm will receive a recorded music intervention prior to and during surgery and postoperatively the first three days (see figure 1). The music intervention prior to surgery is 30 minutes long and takes place before the administration of premedication. After induction of general anaesthesia and after final positioning of the patient a headphone with music is applied and will remain during surgery. The headphones will be removed at the recovery unit, when patients are fully awake. The music interventions after surgery are each 30 minutes long and will take place twice a day, in the morning and evening. In each hospital, the best times to start the intervention will be established to assure blinding of both the anaesthesiologists and pain specialists. Subjects in the control arm will rest for 30 minutes prior to surgery and the administration of premedication, and wear a headphone without music during surgery. After surgery, they will receive regular postoperative care without music interventions. Subjects in the control group are instructed to refrain from much listening to music during the hospital stay. The subjects in both control group and intervention group are requested to self-document all activities performed, such as listening to music, playing video games, using the computer and watching movies and television. Participation ends at the scheduled postoperative check up at the outpatient clinic (see figure 1). All study measurements will take place during hospitalization and at the outpatient clinic. No extra visits to the hospital are required.

**Music selection**

It has been suggested that individual music preference is important to the effect of a music intervention.(22) Nevertheless, a study has shown that playing music from a preselected playlist by the researcher has the largest beneficial effect on postoperative pain, compared to the subject's own favourite music or preselected music without taking the music preference of the subject into account.(19) However, definite conclusions in this regard cannot be drawn. Furthermore, research in rodents suggests that loud rock music may have a negative effect and may act as a stressor.(23)

Therefore, in collaboration with a specialized music therapist we have composed three music playlists without loud rock music, which the subject can choose from. The playlists are categorized into three different genres of music: pop, lounge, and classical music. Subjects can choose from either of these playlists. Subjects may choose a different



For peer review only - http://bmjopen.bmj.com/site/about/guidelines.xhtml

playlist during surgery. Music will be heard through an on-ear headphone connected to a digital music player. After surgery, subjects in the intervention group may listen to their own preferred music. Approval from Buma/Stemra, the Dutch collecting society for composers and music publishers, has been received to use any licensed music.

**Anaesthetic treatment**

There is no nationwide standard anaesthesia protocol for the Nuss-procedure in the Netherlands. Therefore anaesthesia protocols differ between centres. Randomization should control for such variation between centres. However it will be analysed statistically.

All centres apply EMLA cream® at the intravenous line insertion site. Furthermore, all patients receive epidural analgesia, which are preferably placed at 5th thoracic level. All centres used long-acting local anaesthetics with an adjuvant epidurally. General anaesthesia was induced and maintained by propofol combined with opioids and neuromuscular relaxation induced by rocuronium. Postoperative analgesia was maintained with a continuous epidural infusion of a long-acting local aesthetic (ropivacaine 0.2% or bupivacaine 0.125%) with an adjuvant. All patients received weight-based doses of paracetamol and an NSAID postoperatively. After epidural removal pain was treated by oral opioids as required.

However, there are some major differences between hospitals: The Emma Children's hospital gives standard premedication with clonidine 150 microgr and 300 mg gabapentine, while the other two hospitals do not give any pharmacological premedication. Furthermore, in Emma Children's hospital patients receive gabapentin 300 mg twice daily for 5 days and receive patient-controlled analgesia with morphine in addition to the epidural catheters. Finally, while Juliana and Sophia Children's hospitals use sufentanil 0.5 microgr/ml as an epidural adjuvant, Emma Children's hospital uses clonidine 1 microgr/ml.

**Outcome parameters**

The primary outcome parameter is pain, defined as the average pain score, as measured by a Visual Analogue Scale (VAS-pain), that patients will report at the third day postoperatively. The scale of the VAS-pain varies from 0 to 100, whereas 0 is defined as no pain and 100 as the worst pain imaginable. This scale has been recommended and



For peer review only - http://bmjopen.bmj.com/site/about/guidelines.xhtml

validated for the measurement of acute pain in children 8 years of age and above and is also sensitive to changes in pain levels postoperatively. (24-26)

Secondary outcome parameters include:

- The morphine consumption in the first three days postoperative as calculated by the Morphine Equivalent Dose Daily/ kilogram (MEDD/kg) and the consumption of other analgesics in milligrams.
- Physiological variables such as heart rate, blood pressure and respiratory rate will be measured throughout their hospital stay.
- Levels of anxiety and distress will be measured before surgery through the State-Trait-Anxiety Inventory for children. This questionnaire consists of two separate, 20-item, self-report rating scales for measuring trait and state anxiety. The trait anxiety is a relatively stable personality disposition, while state anxiety is the situation-related anxiety and this may differ depending on the stress of a particular moment. (27) The questionnaire has been translated into Dutch and has been validated. (28)
- Quality of life will be measured before surgery and at their first check-up at the outpatient clinic through the Child Health Utility Questionnaire (CHU9D). This validated questionnaire consists of 9 items that assess the child's functioning across domains of worry, sadness, pain, tiredness, annoyance, schoolwork/homework, sleep, problems with daily routine, and ability to join in activities. (29-31)
- Postoperative complications and length of hospital stay are recorded.
- The subject's post-procedural pain after three months will be evaluated with the VAS, the CHU9D and the 'TNO questionnaire for sport and physical activity'. This validated Dutch questionnaire assessed a person's daily activities. (32) This questionnaire serves to measure rehabilitation as a derivative of the post-procedural pain. Baseline measurements for the 'TNO questionnaire for sport and physical activity' will also be performed before surgery.
- Considering the potential influence of pain and the use of analgesics on length of stay, cost-effectiveness of the intervention will be determined through a cost-utility analysis.



For peer review only - http://bmjopen.bmj.com/site/about/guidelines.xhtml

**Eligibility criteria**

Potential subjects visiting the outpatient clinic of the three paediatric surgery departments involved will be informed about our study. A member of the research team undertakes the initial screening for eligibility. The following inclusion criteria apply:

- Age 12 – 18 years
- Scheduled for primary PE repair according to the Nuss-procedure with either one or multiple bars
- Postoperatively, initial placement of a thoracic epidural or both thoracic epidural and patient-controlled analgesia system
- Good knowledge of the Dutch language, by both patients and parents
- Written informed consent. Additional written informed consent by parents or legal guardian is only necessary for children under the age of 16 years.

The following exclusion criteria apply:

- Hearing impairment
- Secondary PE surgery or other prior thoracic surgery
- Known severe mental or psychiatric disorder
- Known impaired communication with patient and parents as collected
- Presence of chronic pain syndrome: ongoing pain lasting longer than 3 months or ongoing pain lasting longer than the reasonably expected healing time for the involved tissues)

One week after being informed about the study, eligible subjects will be called by telephone to inquire if they wish to participate.

**Sample size**

A power calculation was performed by department of Biostatistics of the Erasmus Medical Centre for the primary outcome parameter: pain, defined as the average pain score, as measured by a Visual Analogue Scale (VAS), that patients will report at the third day postoperatively. Evidence on the effects of recorded music interventions prior, during and after surgery in PE repair is lacking. However, a recent meta-analysis, which investigated music interventions on pain in surgical patients, found an overall effect size measured as the Cohen's delta of -0.50 (CI -0.66;-0.34).(19)



For peer review only - http://bmjopen.bmj.com/site/about/guidelines.xhtml

We assumed a low correlation between the VAS score preoperatively and postoperatively of 0.3. Thus, to obtain a power of 90% using a two-sided significance level of $p<0.05$, each study arm requires 77 subjects. To account for dropouts, we will include 85 subjects per study arm, resulting in a total sample size of 170.

**Statistical analysis**

The main study endpoint will be the VAS-pain score reported by the subject during the length of hospital stay, three times a day. The mean VAS-pain score of each day will be calculated per subject. The mean VAS-pain scores between the music and control group on day 3 will be compared with an ANCOVA test, with adjustment for the effects of centre and baseline VAS-pain score. The main analysis will be based on the intention-to-treat principle. In case of non-compliance, a sensitivity analysis will be performed using per-protocol analyses. A two sided p-value of <0.05 will be considered to be statistically significant. For the primary outcome parameter, only the available data will be analysed (no imputation of missing data).

In a sensitivity analysis, we will also adjust for possible confounder variables in the linear regression model for the following variables: age, gender, Body Mass Index, and epidural use. Finally, we will also perform a second sensitivity analysis to determine if the effectiveness of the intervention depends on the type of music chosen, by adding these genres as categories to the linear regression model.

The VAS score of each time point will be analysed using a linear mixed model, with the baseline value (observed before surgery), group (control arm or intervention arm), centre, and time point, and the interaction between group and time point as independent variables. Total consumption of analgesics and type of analgesics in milligrams will be added to the analyses. Also an interaction effect of centre and group will be examined, due to variation in anaesthesia protocols in the participating centres. Using information criteria, it will be determined if it is necessary to add a random intercept and/or random slope of time point to this model, to account for the within-subject correlations. If required, a transformation of the outcome will be applied to ensure normality of the model residuals.

The secondary outcome parameters will be analysed as follows.



For peer review only - http://bmjopen.bmj.com/site/about/guidelines.xhtml

• Morphine equivalence daily dose/kilogram (MEDD/kg) and total dosage of other analgesia:

There may be differences between centres in usage of patient controlled analgesia and epidural anaesthesia. Therefore, the difference between the intervention group and the control group will be tested using multiple linear regression, with adjustment for the effects of centre. When necessary, an appropriate transformation of the outcome (MEDD/kg) or total dosage of other analgesia in milligrams will be performed to achieve a normal distribution of the residuals.

• Score on State-Trait-Anxiety-Inventory questionnaire (STAI) and Health related quality of life (HRQoL):

The scores of the STAI and HRQoL questionnaires will be compared between groups using analysis of covariance, with group, centre, and the baseline STAI score and HRQoL score before the intervention or the resting period as independent variables.

• Physiologic measurements, including blood pressure, heart rate and respiratory rate:

These variables will be analysed using a linear mixed model, with the baseline value (observed before surgery), group (control arm or intervention arm), centre, time point, and the interaction between group and time point as independent variables.

Using information criteria, it will be determined if it is necessary to add to a random intercept and/or random slope of time point to this model, to account for the within subject correlations. If necessary, a transformation of the outcome will be applied to ensure normality of the model residuals.

• Complications, like post-operative ileus (n of days), nausea/vomiting (*n* of days and also anti-emetics used), pruritus:

The duration of post-operative ileus, nausea, and vomiting will be compared between groups using a Mann-Whitney test, stratified by centre (i.e. a Van Elteren test). The percentage of patients with pruritus will be compared between groups using a stratified chi-square test.

• Length of hospital stay (n of days):

The length of hospital stay will be compared between groups using a Mann-Whitney test, stratified by centre (i.e. a Van Elteren test).

**Economic evaluation**



For peer review only - http://bmjopen.bmj.com/site/about/guidelines.xhtml

We will analyse the cost-effectiveness of the music intervention versus 'standard care' from a health care perspective, using the techniques of cost-effectiveness analysis and cost-utility analysis and following recommended methods for economic evaluations. (33)

Medical costs (i.e., costs within the health care sector) will be analysed, including costs of surgeries, hospital days (on the ward or ICU), medications (such as analgesics), diagnostic radiography, and intercollegiate consultations. For the intervention group, costs of the music intervention will be added, mainly consisting of a Spotify™ subscription. In addition, costs of health care use after the initial hospitalization will be calculated (e.g., outpatient visits, consultations by telephone, (pain) medication, and rehabilitation). Resource consumption for all these items will be derived from electronic databases at the participating centres and from a questionnaire (based on the iMTA Medical Consumption Questionnaire). (34) Unit prices (calculated using economic cost prices or standard prices) will be multiplied by the quantities for each resource used, and then summed over the separate types of resource to give a total cost per patient. Non-medical costs (e.g., out-of-pocket costs and costs of productivity losses incurred by the parents) will be ignored in this study, as these are expected to be relatively minor and not to differ notably between the study groups.

Regarding the patient outcomes, the economic evaluation will look at pain (as measured by the VAS) and HRQoL measured by the CHU9D. The CHU9D is a preference-based measure of HRQoL allowing for the calculation of QALYs, which is a commonly used health outcome measure to calculate the benefits of new interventions within cost-utility analyses for economic evaluation. QALYs will be calculated based on the CHU9D and using linear interpolation between measurement points.

Building on these data on costs and patient outcomes, incremental cost-effectiveness ratios (ICERs) will be calculated, expressed as incremental costs to obtain a reduction of 1 additional unit (10 mm or 1 cm) in the VAS score and as incremental costs per QALY gained. Otherwise, the economic evaluation will focus on dominance of one treatment over the other with respect to lower cost and greater effect. The time horizon of the analysis will be the 3-months follow-up period (starting at the beginning of the hospital



For peer review only - http://bmjopen.bmj.com/site/about/guidelines.xhtml

admission for the PE repair). As a consequence, discounting will not be necessary. Analysis of uncertainty is illustrated through cost-effectiveness planes (via bootstrapping). Sensitivity analysis will be performed to assess the robustness of the analysis to certain assumptions.

**Trial monitoring**

An independent trial monitor has been appointed to oversee all aspects of design, delivery, quality assurance and data analysis. The trial will be monitored at least once per year.

**Data management**

Participant data are stored on a secure database in accordance with the General Data Protection Regulations (2018). Data are handled confidentially, de-identified and coded with a unique study number. Published data from this study cannot be traced to a specific subject. Data management for the study was done through OpenClinica and LimeSurvey. Study staff assigned to manage data has access to the OpenClinica and LimeSurvey application and is required to login via an individualized username and password combination. Study staff located at other institutions only has access to the data collected at their sites. The local investigators will safeguard the key that links the unique study number to the patients name at a separate server.

Trial documentation and data will be archived for at least 10 years after completion of the trial.

**Patient and public involvement statement**

Patients undergoing PE repair prior to the start of this study evaluated and helped us composing our preselected music playlists.



For peer review only - http://bmjopen.bmj.com/site/about/guidelines.xhtml

## *Ethics and dissemination*

### Ethics

This study protocol has been reviewed and approved by the medical ethics review board at the Erasmus Medical Centre, in Rotterdam on 5 September 2018. This study is being conducted according to the principles of the Declaration of Helsinki (64$^{th}$ WMA General Assembly, Fortaleza, Brazil, October 2013) and in accordance with the Medical Research Involving Human Subject Act (Dutch: WMO). The trial is registered with the Netherlands Trial Register NL6863. To prohibit playing music on the operating room and testing the epidural sensory block daily were approved and implemented as a minor amendment on 9 October 2019.

### Benefits and risks assessment, group relatedness

There are no risks associated with listening to music, except potential hearing damage. To prevent hearing damage, the music administered on the headphones will be adjusted to a maximum of 60 dB, which is the advised loudness of a music intervention in medical care.(35) The maximum dB advised to be exposed to for forty hours a week is 80 dB. (36) Therefore, risk of participation can be considered negligible and the burden minimal. During the informed consent process, it will be made clear that participation in this study has no direct benefits to the patient, and that refusal to participate will not have impact on the care received by any of the medical staff. PE is preferably corrected at age 12-18. This study therefore cannot be conducted without the participation of this group.

Music intervention itself is considered harmless and safe. Therefore, we expect no intervention-related serious adverse events or any other disadvantages for participants in this study.

### Dissemination

The research team is committed to full disclosure of the results of the trial. Findings will be reported in accordance with CONSORT guidelines and we aim to publish in high impact journals. Given the multitude of outcome parameters, results will be divided over several papers. The funder will take no role in the analysis or interpretation of trial results.



For peer review only - http://bmjopen.bmj.com/site/about/guidelines.xhtml

**Authors' contribution:** Each author has contributed significantly to, and is willing to take public responsibility for, one or more aspects of the study. All authors have seen and approved the final version of the manuscript being submitted. The article is the authors' original work, has not received prior publication and is not under consideration for publication elsewhere.

**Conflicts of interest:** none declared.

**Funding:** This research is funded by the Erasmus Medical Centre, Rotterdam, the Netherlands.

**Patient consent:** Not required

**Ethical approval:** The study protocol has received ethical approval by the medical ethical review committee at the Erasmus Medical Centre, in Rotterdam prior to the beginning of the study.

For peer review only



For peer review only - http://bmjopen.bmj.com/site/about/guidelines.xhtml

## References

1. Lam MW, Klassen AF, Montgomery CJ, LeBlanc JG, Skarsgard ED. Quality-of-life outcomes after surgical correction of pectus excavatum: a comparison of the Ravitch and Nuss procedures. J Pediatr Surg. 2008;43(5):819-25.
2. Kelly RE, Jr., Cash TF, Shamberger RC, Mitchell KK, Mellins RB, Lawson ML, et al. Surgical repair of pectus excavatum markedly improves body image and perceived ability for physical activity: multicenter study. Pediatrics. 2008;122(6):1218-22.
3. Frantz FW. Indications and guidelines for pectus excavatum repair. Curr Opin Pediatr. 2011;23(4):486-91.
4. Fonkalsrud EW. Current management of pectus excavatum. World J Surg. 2003;27(5):502-8.
5. Mao YZ, Tang S, Li S. Comparison of the Nuss versus Ravitch procedure for pectus excavatum repair: an updated meta-analysis. J Pediatr Surg. 2017;52(10):1545-52.
6. Abid I, Ewais MM, Marranca J, Jaroszewski DE. Pectus Excavatum: A Review of Diagnosis and Current Treatment Options. J Am Osteopath Assoc. 2017;117(2):106-13.
7. Dean C, Etienne D, Hindson D, Matusz P, Tubbs RS, Loukas M. Pectus excavatum (funnel chest): a historical and current prospective. Surg Radiol Anat. 2012;34(7):573-9.
8. Fonkalsrud EW, Beanes S, Hebra A, Adamson W, Tagge E. Comparison of minimally invasive and modified Ravitch pectus excavatum repair. J Pediatr Surg. 2002;37(3):413-7.
9. Molik KA, Engum SA, Rescorla FJ, West KW, Scherer LR, Grosfeld JL. Pectus excavatum repair: experience with standard and minimal invasive techniques. J Pediatr Surg. 2001;36(2):324-8.
10. Papic JC, Finnell SM, Howenstein AM, Breckler F, Leys CM. Postoperative opioid analgesic use after Nuss versus Ravitch pectus excavatum repair. J Pediatr Surg. 2014;49(6):919-23.
11. St Peter SD, Weesner KA, Weissend EE, Sharp SW, Valusek PA, Sharp RJ, et al. Epidural vs patient-controlled analgesia for postoperative pain after pectus excavatum repair: a prospective, randomized trial. J Pediatr Surg. 2012;47(1):148-53.
12. McCaffrey R, Frock TL, Garguilo H. Understanding chronic pain and the mind-body connection. Holist Nurs Pract. 2003;17(6):281-7; quiz 8-9.
13. Good M. Effects of relaxation and music on postoperative pain: a review. J Adv Nurs. 1996;24(5):905-14.
14. Voss JA, Good M, Yates B, Baun MM, Thompson A, Hertzog M. Sedative music reduces anxiety and pain during chair rest after open-heart surgery. Pain. 2004;112(1-2):197-203.
15. Nguyen TN, Nilsson S, Hellström AL, Bengtson A. Music therapy to reduce pain and anxiety in children with cancer undergoing lumbar puncture: A randomized clinical trial. J Pediatr Oncol Nurs. 2010;27(3):146-55.
16. Nilsson U, Rawal N, Enqvist B, Unosson M. Analgesia following music and therapeutic suggestions in the PACU in ambulatory surgery; A randomized controlled trial. Acta Anaesthesiol Scand. 2003;47(3):278-83.
17. Wang Y, Dong Y, Li Y. Perioperative psychological and music interventions in elderly patients undergoing spinal anesthesia: Effect on anxiety, heart rate variability, and postoperative pain. Yonsei Med J. 2014;55(4):1101-5.
18. Klassen JA, Liang Y, Tjosvold L, Klassen TP, Hartling L. Music for pain and anxiety in children undergoing medical procedures: a systematic review of randomized controlled trials. Ambul Pediatr. 2008;8(2):117-28.



For peer review only - http://bmjopen.bmj.com/site/about/guidelines.xhtml

19. Kuhlmann AYR, de Rooij A, Kroese LF, van Dijk M, Hunink MGM, Jeekel J. Meta-analysis evaluating music interventions for anxiety and pain in surgery. Br J Surg. 2018;105(7):773-83.
20. Fu VX, Oomens P, Klimek M, Verhofstad MHJ, Jeekel J. The Effect of Perioperative Music on Medication Requirement and Hospital Length of Stay: A Meta-analysis. Ann Surg. 2019.
21. van der Heijden MJ, Oliai Araghi S, Jeekel J, Reiss IK, Hunink MG, van Dijk M. Do Hospitalized Premature Infants Benefit from Music Interventions? A Systematic Review of Randomized Controlled Trials. PLoS One. 2016;11(9):e0161848.
22. Kaempf G, Amodei ME. The effect of music on anxiety. A research study. AORN J. 1989;50(1):112-8.
23. Kuhlmann A. Music seems to affect rodents: A systematic review of experimental research. 2017.
24. McGrath PJ, Walco GA, Turk DC, Dworkin RH, Brown MT, Davidson K, et al. Core outcome domains and measures for pediatric acute and chronic/recurrent pain clinical trials: PedIMMPACT recommendations. J Pain. 2008;9(9):771-83.
25. Huguet A, Stinson JN, McGrath PJ. Measurement of self-reported pain intensity in children and adolescents. J Psychosom Res. 2010;68(4):329-36.
26. Tyler DC, Tu A, Douthit J, Chapman CR. Toward validation of pain measurement tools for children: a pilot study. Pain. 1993;52(3):301-9.
27. Moerman N, van Dam FS, Muller MJ, Oosting H. The Amsterdam Preoperative Anxiety and Information Scale (APAIS). Anesth Analg. 1996;82(3):445-51.
28. Ploeg HMvd DP, Spielberger CD, Defares PB, Spielberger CD. Handleiding bij de Zelf-Beoordelings Vragenlijst ZBV : een nederlandstalige bewerking van de Spielberger State- Trait Anxiety Inventory STAI-DY. Lisse: Swets & Zeitlinger. 1980.
29. Stevens K. Valuation of the Child Health Utility 9D Index. Pharmacoeconomics. 2012;30(8):729-47.
30. Stevens K, Ratcliffe J. Measuring and valuing health benefits for economic evaluation in adolescence: an assessment of the practicality and validity of the child health utility 9D in the Australian adolescent population. Value Health. 2012;15(8):1092-9.
31. Ratcliffe J, Stevens K, Flynn T, Brazier J, Sawyer M. An assessment of the construct validity of the CHU9D in the Australian adolescent general population. Qual Life Res. 2012;21(4):717-25.
32. Ooijendijk. Advies Consensus Vragenlijsten Sport en Bewegen. 2007.
33. National Health Care Institute. Guidelines for economic evaluations of health care [in Dutch]. National Health Care Institute, Diemen. 2015.
34. Bouwmans C H-vRL, Koopmanschap M, Krol M, Severens H, Brouwer W iMTA Medical Consumption Questionnaire. Institute for Medical Technology Assessment. Erasmus Universiteit Rotterdam, Rotterdam. 2013.
35. Nilsson U. The anxiety- and pain-reducing effects of music interventions: a systematic review. AORN J. 2008;87(4):780-807.
36. Marinus E, Alphen WJTv. Geluid en trillingen. Zeist: Kerckebosch Uitgeverij|Studiecentrum; 2013.

For peer review only

**Figure legends**

Figure 1 Flow chart of study interventions and assessments

For peer review only



For peer review only - http://bmjopen.bmj.com/site/about/guidelines.xhtml

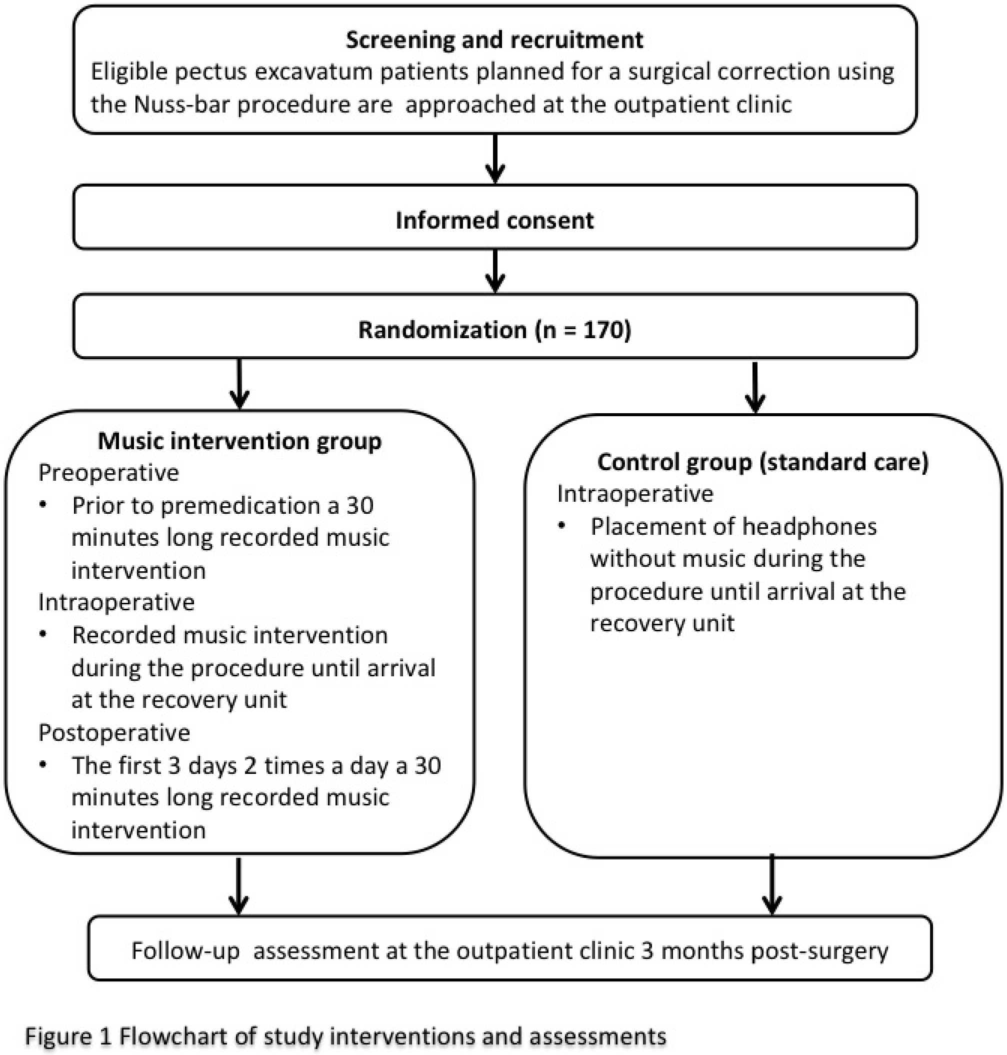


Figure 1 Flowchart of study interventions and assessments.

234x250mm (72 x 72 DPI)

STANDARD PROTOCOL ITEMS: RECOMMENDATIONS FOR INTERVENTIONAL TRIALS

SPIRIT 2013 Checklist for the IMPECT trial (Interventions with Music in Pectus Excavatum Treatment): Recommended items to address in a clinical trial protocol and related documents*

| Section/item | Item No | Description | Addressed on page number in original protocol |
|---|---|---|---|
| **Administrative information** | | | |
| Title | 1 | Descriptive title identifying the study design, population, interventions, and, if applicable, trial acronym | 1 |
| Trial registration | 2a | Trial identifier and registry name. If not yet registered, name of intended registry | 41 |
| | 2b | All items from the World Health Organization Trial Registration Data Set | Yes, www.trialregister.nl |
| Protocol version | 3 | Date and version identifier | 1 |
| Funding | 4 | Sources and types of financial, material, and other support | 5 |
| Roles and responsibilities | 5a | Names, affiliations, and roles of protocol contributors | 2, 3, 4 |
| | 5b | Name and contact information for the trial sponsor | 5 |
| | 5c | Role of study sponsor and funders, if any, in study design; collection, management, analysis, and interpretation of data; writing of the report; and the decision to submit the report for publication, including whether they will have ultimate authority over any of these activities | 33, 40, 41 |

For peer review only

| | | | |
|---|---|---|---|
| | 5d | Composition, roles, and responsibilities of the coordinating centre, steering committee, endpoint adjudication committee, data management team, and other individuals or groups overseeing the trial, if applicable (see Item 21a for data monitoring committee) | 40 |
| **Introduction** | | | |
| Background and rationale | 6a | Description of research question and justification for undertaking the trial, including summary of relevant studies (published and unpublished) examining benefits and harms for each intervention | 12, 13, 14 |
| | 6b | Explanation for choice of comparators | 13, 15, 18 |
| Objectives | 7 | Specific objectives or hypotheses | 14 |
| Trial design | 8 | Description of trial design including type of trial (eg, parallel group, crossover, factorial, single group), allocation ratio, and framework (eg, superiority, equivalence, noninferiority, exploratory) | 15 |
| **Methods: Participants, interventions, and outcomes** | | | |
| Study setting | 9 | Description of study settings (eg, community clinic, academic hospital) and list of countries where data will be collected. Reference to where list of study sites can be obtained | 15, 18, 19 |
| Eligibility criteria | 10 | Inclusion and exclusion criteria for participants. If applicable, eligibility criteria for study centres and individuals who will perform the interventions (eg, surgeons, psychotherapists) | 16, 17 |
| Interventions | 11a | Interventions for each group with sufficient detail to allow replication, including how and when they will be administered | 18, 19 |
| | 11b | Criteria for discontinuing or modifying allocated interventions for a given trial participant (eg, drug dose change in response to harms, participant request, or improving/worsening disease) | 30, 31 |
| | 11c | Strategies to improve adherence to intervention protocols, and any procedures for monitoring adherence (eg, drug tablet return, laboratory tests) | 18, 19 |
| | 11d | Relevant concomitant care and interventions that are permitted or prohibited during the trial | 18, 19 |



For peer review only - http://bmjopen.bmj.com/site/about/guidelines.xhtml

| | | | |
|---|---|---|---|
| Outcomes | 12 | Primary, secondary, and other outcomes, including the specific measurement variable (eg, systolic blood pressure), analysis metric (eg, change from baseline, final value, time to event), method of aggregation (eg, median, proportion), and time point for each outcome. Explanation of the clinical relevance of chosen efficacy and harm outcomes is strongly recommended | 23, 24, 25, 26, 27, 28, 29, 30 and Table 1 |
| Participant timeline | 13 | Time schedule of enrolment, interventions (including any run-ins and washouts), assessments, and visits for participants. A schematic diagram is highly recommended (see Figure) | 24, 25, 26, 27, 28, 29, 30, Table 1 |
| Sample size | 14 | Estimated number of participants needed to achieve study objectives and how it was determined, including clinical and statistical assumptions supporting any sample size calculations | 17 |
| Recruitment | 15 | Strategies for achieving adequate participant enrolment to reach target sample size | 18 |

**Methods: Assignment of interventions (for controlled trials)**

Allocation:

| | | | |
|---|---|---|---|
| Sequence generation | 16a | Method of generating the allocation sequence (eg, computer-generated random numbers), and list of any factors for stratification. To reduce predictability of a random sequence, details of any planned restriction (eg, blocking) should be provided in a separate document that is unavailable to those who enrol participants or assign interventions | 24 |
| Allocation concealment mechanism | 16b | Mechanism of implementing the allocation sequence (eg, central telephone; sequentially numbered, opaque, sealed envelopes), describing any steps to conceal the sequence until interventions are assigned | 24 |
| Implementation | 16c | Who will generate the allocation sequence, who will enrol participants, and who will assign participants to interventions | 24 |
| Blinding (masking) | 17a | Who will be blinded after assignment to interventions (eg, trial participants, care providers, outcome assessors, data analysts), and how | 19, 24 |
| | 17b | If blinded, circumstances under which unblinding is permissible, and procedure for revealing a participant’s allocated intervention during the trial | NA |

**Methods: Data collection, management, and analysis**

| | | | |
|---|---|---|---|
| Data collection methods | 18a | Plans for assessment and collection of outcome, baseline, and other trial data, including any related processes to promote data quality (eg, duplicate measurements, training of assessors) and a description of study instruments (eg, questionnaires, laboratory tests) along with their reliability and validity, if known. Reference to where data collection forms can be found, if not in the protocol | 18, 19, 24, 25, 26, 27, 28, 29, 30 and Table 1 |
| | 18b | Plans to promote participant retention and complete follow-up, including list of any outcome data to be collected for participants who discontinue or deviate from intervention protocols | 19, 30, 31 |
| Data management | 19 | Plans for data entry, coding, security, and storage, including any related processes to promote data quality (eg, double data entry; range checks for data values). Reference to where details of data management procedures can be found, if not in the protocol | 40 |
| Statistical methods | 20a | Statistical methods for analysing primary and secondary outcomes. Reference to where other details of the statistical analysis plan can be found, if not in the protocol | 34, 35, 36, 37 |
| | 20b | Methods for any additional analyses (eg, subgroup and adjusted analyses) | 34, 35, 36 |
| | 20c | Definition of analysis population relating to protocol non-adherence (eg, as randomised analysis), and any statistical methods to handle missing data (eg, multiple imputation) | 15 |
| **Methods: Monitoring** | | | |
| Data monitoring | 21a | Composition of data monitoring committee (DMC); summary of its role and reporting structure; statement of whether it is independent from the sponsor and competing interests; and reference to where further details about its charter can be found, if not in the protocol. Alternatively, an explanation of why a DMC is not needed | 17, 18 |
| | 21b | Description of any interim analyses and stopping guidelines, including who will have access to these interim results and make the final decision to terminate the trial | NA |
| Harms | 22 | Plans for collecting, assessing, reporting, and managing solicited and spontaneously reported adverse events and other unintended effects of trial interventions or trial conduct | 32, 33, 40 |
| Auditing | 23 | Frequency and procedures for auditing trial conduct, if any, and whether the process will be independent from investigators and the sponsor | 40 |
| **Ethics and dissemination** | | | |

| | | | |
|---|---|---|---|
| Research ethics approval | 24 | Plans for seeking research ethics committee/institutional review board (REC/IRB) approval | Is stated in cover letter to REC as required in the Netherlands |
| Protocol amendments | 25 | Plans for communicating important protocol modifications (eg, changes to eligibility criteria, outcomes, analyses) to relevant parties (eg, investigators, REC/IRBs, trial participants, trial registries, journals, regulators) | 40, 41 |
| Consent or assent | 26a | Who will obtain informed consent or assent from potential trial participants or authorised surrogates, and how (see Item 32) | 15, 38 |
| | 26b | Additional consent provisions for collection and use of participant data and biological specimens in ancillary studies, if applicable | NA |
| Confidentiality | 27 | How personal information about potential and enrolled participants will be collected, shared, and maintained in order to protect confidentiality before, during, and after the trial | 24, 40 |
| Declaration of interests | 28 | Financial and other competing interests for principal investigators for the overall trial and each study site | 39 |
| Access to data | 29 | Statement of who will have access to the final trial dataset, and disclosure of contractual agreements that limit such access for investigators | 40, 41, disclosure of contractual agreements at request |
| Ancillary and post-trial care | 30 | Provisions, if any, for ancillary and post-trial care, and for compensation to those who suffer harm from trial participation | 39 |
| Dissemination policy | 31a | Plans for investigators and sponsor to communicate trial results to participants, healthcare professionals, the public, and other relevant groups (eg, via publication, reporting in results databases, or other data sharing arrangements), including any publication restrictions | 41 |
| | 31b | Authorship eligibility guidelines and any intended use of professional writers | 40, 41, In contractual agreements |



For peer review only - http://bmjopen.bmj.com/site/about/guidelines.xhtml

| | | | |
|---|---|---|---|
| | 31c | Plans, if any, for granting public access to the full protocol, participant-level dataset, and statistical code | NA |
| **Appendices** | | | |
| Informed consent materials | 32 | Model consent form and other related documentation given to participants and authorised surrogates | Available on request |
| Biological specimens | 33 | Plans for collection, laboratory evaluation, and storage of biological specimens for genetic or molecular analysis in the current trial and for future use in ancillary studies, if applicable | NA |

*It is strongly recommended that this checklist be read in conjunction with the SPIRIT 2013 Explanation & Elaboration for important clarification on the items. Amendments to the protocol should be tracked and dated. The SPIRIT checklist is copyrighted by the SPIRIT Group under the Creative Commons "Attribution-NonCommercial-NoDerivs 3.0 Unported" license.

For peer review only

| Data category | Information |
|---|---|
| Primary registry and trial identifying number | Dutch Trial Registry, NL6863 |
| Date of registration in primary registry | 22 February, 2018 |
| Secondary identifying numbers | NTR7041 |
| Source(s) of monetary or material support | Erasmus University Medical Centre |
| Primary sponsor | Erasmus University Medical Centre |
| Secondary sponsor(s) | NA |
| Contact for public queries | RJ Billar, MD, (phone: 0031646794742, e-mail: r.billar@erasmusmc.nl) |
| Contact for scientific queries | RJ Billar, MD, (phone: 0031646794742, e-mail: r.billar@erasmusmc.nl)<br>Erasmus University Medical Centre, the Netherlands |
| Public title | Music interventions in operative treatment of funnel chest |
| Scientific title | Music interventions in pectus excavatum treatment (IMPECT trial) |
| Countries of recruitment | The Netherlands |
| Health condition(s) or problem(s) studied | Pectus Excavatum |
| Intervention(s) | Active comparator: recorded music interventions before, during and the first 3 days after operative repair of pectus excavatum<br>Placebo comparator: No music |
| Key inclusion and exclusion criteria | **Inclusion criteria**<br>▪ Age 12 – 18 years |

| | |
|---|---|
| | ▪ Scheduled for primary pectus excavatum repair<br><br>▪ Surgery according to the Nuss-procedure<br><br>▪ Repair with either one or multiple bars<br><br>▪ General anaesthesia during surgery<br><br>▪ Placement of thoracic epidural or both thoracic epidural and patient controlled analgesia system<br><br>▪ Good knowledge of Dutch or English language by patient and parents<br><br>▪ Written informed consent<br><br>**Exclusion criteria**<br>Hearing impairments<br><br>▪ Secondary pectus excavatum surgery<br><br>▪ Other prior thoracic surgery<br><br>▪ No thoracic epidural<br><br>▪ Severe mental or psychiatric disorder<br><br>▪ Impaired communication with patient and parents<br><br>▪ Missing informed consent<br><br>▪ Presence of chronic pain syndrome (defined as ongoing pain lasting longer than 3 months or ongoing pain lasting longer than the reasonable expected healing time for the involved tissues) |
| Study type | Interventional<br>Allocation: randomized<br>Intervention model: two arms, parallel assignment<br>Masking: single blind (anaesthesiologists, statistician) |



For peer review only - http://bmjopen.bmj.com/site/about/guidelines.xhtml

| | |
|---|---|
| | Purpose: prevention |
| Date of first enrolment | January 2019 |
| Target sample size | 170 |
| Recruitment status | Recruiting |
| Primary outcome(s) | Pain measured by the Visual Analogue scale |
| Key secondary outcomes | Anxiety (measured by the state-trait anxiety inventory), vital parameters, (health related) quality of life, rehabilitation, medication use, complication, patient satisfaction, length of hospital stay, costs |

For peer review only

BMJ Open

# Interventions with Music in Pectus Excavatum Treatment (IMPECT trial): A study protocol for a randomized controlled trial investigating the clinical effects of perioperative music interventions



| | |
|---|---|
| Journal: | *BMJ Open* |
| Manuscript ID | bmjopen-2019-036380.R1 |
| Article Type: | Protocol |
| Date Submitted by the Author: | 16-Mar-2020 |
| Complete List of Authors: | Billar, Ryan; Erasmus MC Sophia Children Hospital, Pediatric Surgery<br>Kühlmann, Rosalie; Saint Antonius Hospital, Anaesthesiology<br>Schnater, Marco; Erasmus MC Sophia Children Hospital, Paediatric Surgery<br>Vlot, John; Erasmus MC Sophia Children Hospital, Paediatric Surgery<br>Tomas, Jeremy; Erasmus MC Sophia Children Hospital, Anaesthesiology<br>Zijp, Gerda; Haga Hospital Juliana Childrens Hospital, Paediatric Surgery<br>Rad, Mandana; Haga Hospital Juliana Childrens Hospital, Anaesthesiology<br>de Beer, Sjoerd; Emma Childrens Hospital AMC, Paediatric Surgery<br>Stevens, Markus; Emma Childrens Hospital AMC, Anaesthesiology<br>Poley, Marten; Erasmus University Rotterdam, Institute for Medical Technology Assessment; Erasmus MC Sophia Children Hospital,<br>van Rosmalen, Joost; Erasmus MC, Biostatistics<br>Jeekel, Johannes; Erasmus MC,<br>Wijnen, Rene; Erasmus MC Sophia Children Hospital, Paediatric Surgery |
| <b>Primary Subject Heading</b>: | Surgery |
| Secondary Subject Heading: | Anaesthesia, Paediatrics |
| Keywords: | Paediatric thoracic surgery < PAEDIATRIC SURGERY, Paediatric anaesthesia < PAEDIATRICS, PAIN MANAGEMENT |
| | |



SCHOLARONE™
Manuscripts

*I, the Submitting Author has the right to grant and does grant on behalf of all authors of the Work (as defined in the below author licence), an exclusive licence and/or a non-exclusive licence for contributions from authors who are: i) UK Crown employees; ii) where BMJ has agreed a CC-BY licence shall apply, and/or iii) in accordance with the terms applicable for US Federal Government officers or employees acting as part of their official duties; on a worldwide, perpetual, irrevocable, royalty-free basis to BMJ Publishing Group Ltd ("BMJ") its licensees and where the relevant Journal is co-owned by BMJ to the co-owners of the Journal, to publish the Work in this journal and any other BMJ products and to exploit all rights, as set out in our licence.*

*The Submitting Author accepts and understands that any supply made under these terms is made by BMJ to the Submitting Author unless you are acting as an employee on behalf of your employer or a postgraduate student of an affiliated institution which is paying any applicable article publishing charge ("APC") for Open Access articles. Where the Submitting Author wishes to make the Work available on an Open Access basis (and intends to pay the relevant APC), the terms of reuse of such Open Access shall be governed by a Creative Commons licence – details of these licences and which Creative Commons licence will apply to this Work are set out in our licence referred to above.*

*Other than as permitted in any relevant BMJ Author's Self Archiving Policies, I confirm this Work has not been accepted for publication elsewhere, is not being considered for publication elsewhere and does not duplicate material already published. I confirm all authors consent to publication of this Work and authorise the granting of this licence.*

For peer review only

# Interventions with Music in PECTus excavatum treatment (IMPECT trial): A study protocol for a randomized controlled trial investigating the clinical effects of perioperative music interventions

RJ Billar[1], AYR. Kühlmann[1,10], JM Schnater[1], J Vlot[1], JJP Tomas[2], GW Zijp[6], M Rad[7], SA de Beer[8], MF Stevens[9], MJ Poley[1,5], J van Rosmalen[3], JF Jeekel[4], RMH Wijnen[1]

**Author affiliations**

Erasmus University Medical Centre – Sophia Children's Hospital, Rotterdam, the Netherlands:

1 department of Paediatric Surgery; 2 department of Anaesthesiology

Erasmus University Medical Centre, Rotterdam, the Netherlands:

3 department of Biostatistics; 4 department of Neuroscience

Erasmus University, Rotterdam, the Netherlands:

5 institute for Medical Technology Assessment

Haga Hospital – Juliana Children's Hospital, the Hague, the Netherlands:

6 department of Surgery; 7 department of Anaesthesiology

Academic Medical Centre - Emma Children's Hospital, Amsterdam, the Netherlands:

8 department of Surgery; 9 department of Anaesthesiology

St Antonius Hospital, Nieuwegein, The Netherlands:

10 department of Anaesthesiology

Corresponding author: RJ Billar

Department of paediatric surgery

Erasmus University Medical Centre-Sophia Children's Hospital

Doctor Molewaterplein 40, 3015GD Rotterdam, the Netherlands

Phone: 00310-7036783

Fax: 00310-7036802

Email: r.billar@erasmusmc.nl

For peer review only

## Abstract

**Introduction:** Pectus excavatum repair is associated with substantial postoperative pain, despite the use of epidural analgesia and other analgesic regimens. Perioperative recorded music interventions have been shown to alleviate pain and anxiety in adults, but evidence for children and adolescents is still lacking. This study protocol describes a randomized controlled trial that evaluates effects of recorded music interventions on postoperative pain relief in in children and adolescents after pectus excavatum repair.

**Methods:**

A multicentre, randomized controlled trial was set up comparing the effects of perioperative recorded music interventions in addition to standard care with those of standard care only in patients undergoing a Nuss-procedure for pectus excavatum repair. One hundred and seventy subjects (12-18 years of age) will be included in three centres in the Netherlands. Patient inclusion has started in November 2018, and is ongoing. The primary outcome is self-reported perceived pain measured on the Visual Analogue Scale. Secondary outcomes are anxiety level, analgesics consumption, vital parameters such as heart rate, blood pressure and respiratory rate, length of hospital stay, postoperative complications, quality of life, and cost-effectiveness.

**Ethics and dissemination**

This study is being conducted in accordance with the Declaration of Helsinki. The medical ethics review board of Erasmus University Medical Centre Rotterdam, the Netherlands, has approved this protocol. Results will be disseminated via peer-reviewed scientific journals and conference presentations.

**Trial registration number in the Dutch Trial Registration** NL6863




For peer review only - http://bmjopen.bmj.com/site/about/guidelines.xhtml

**Strengths and limitations**

- This study is the first multicentre randomized controlled trial evaluating the effects of recorded music interventions on pain experience in older children before, during and after pectus excavatum repair with the Nuss-procedure.
- Data will be collected during hospitalization and up until 3 months postoperatively to shed light on the effect of perioperative recorded music interventions during hospitalization and after hospitalization evaluating both short-term and potentially long-term effects.
- The study participants and participating surgeons are not blinded to the interventions, which is a limitation; however, the anaesthesiologists and pain specialists will be blinded to the study arm allocation.

## *Introduction*

Pectus excavatum (PE) is the most common congenital chest wall deformity affecting 0.1-0.8% of live births, affecting boys more than girls. Operative repair is indicated when symptoms or signs of heart and/or lung dysfunction are present,(1) or when the patient is much concerned about the cosmetic appearance and psychosocial problems occur.(2, 3) The optimal age for repair is between 12 and 16 years.(4) Numerous surgical techniques have been developed to correct PE, of which the Nuss-procedure is now among the most commonly employed techniques. (5, 6) The Nuss-procedure involves inserting a convex steel bar beneath the sternum, to reposition the sternum anteriorly and thereby effectively correcting the deformity.(7) It is associated, however, with substantial postoperative pain, despite the use of epidural analgesia or patient-controlled intravenous opioid administration.(8, 9) Pain management is the critical component of postoperative care as postoperative pain has implications for activity and quality of life (10) and is the primary factor determining the length of hospital stay.(11) Therefore, interest is growing in finding new ways to alleviate postoperative pain, such as perioperative music interventions. In previous studies in adult surgical patients, recorded music interventions reduce pain medication consumption and improve the management of pain and anxiety.(12-20) However in children and adolescents undergoing surgery, a definite conclusion about the effect of recorded music interventions has yet to be drawn.(21) Especially in paediatric surgical procedures associated with substantial postoperative pain, such as the Nuss-procedure, music



For peer review only - http://bmjopen.bmj.com/site/about/guidelines.xhtml

interventions might be effective in reducing children's pain and anxiety. We designed a multicentre, randomized controlled trial (IMPECT trial) to evaluate whether adjuvant recorded music interventions are indeed associated with less postoperative pain in children and adolescents undergoing the Nuss-procedure for PE repair.

## *Methods and analysis*

### Study design

The IMPECT trial is a randomized controlled trial with two study arms, designed to compare the effects on postoperative pain of perioperative recorded music interventions in addition to standard care (intervention group) versus standard care (control group) – prior, during and after the Nuss-procedure for PE repair. We will include one hundred and seventy subjects children and adolescents (12-18 year of age) operated on in three centres in the Netherlands: the Erasmus University Medical Centre-Sophia Children's Hospital, Rotterdam; Haga Hospital-Juliana Children's Hospital, The Hague; Academic Medical Centre-Emma Children's Hospital, Amsterdam. We started enrolment in November 2018. The first patient included was in January 2019.
This study protocol follows the Standard Protocol Items: Recommendations for Interventional Trials (SPIRIT) guidelines (see SPIRIT checklist in online supplemental files). The underlying protocol follows the Consolidated Standards of Reporting Trials (CONSORT) guidelines for non-pharmacological treatments. This trial was registered on trialregister.nl (NL6863).

### Randomization, blinding and treatment allocation

A parallel randomization with equal allocation ratio is being carried out to individually allocate subjects to either the intervention or the control group. An online web-based randomization program (ALEA; FormVision, Abcoude, the Netherlands) generates the random allocation sequence by the use of random block size randomization and is stratified by centre with an equal allocation-ratio per centre in both study arms. Allocation concealment will be ensured, as the service will not release the randomization code until the patient has been recruited into the trial. The anaesthesiologists and pain specialists involved do not have access to the randomization program and are blinded to the subject's study arm allocation, as well as the person analysing the data.



For peer review only - http://bmjopen.bmj.com/site/about/guidelines.xhtml

## Interventions

Subjects in the intervention arm receive a recorded music intervention prior to and during surgery and postoperatively the first three days (see figure 1). The music intervention prior to surgery is 30 minutes long and takes place before the administration of premedication. After induction of general anaesthesia and after final positioning of the patient a headphone with music is applied and will remain during surgery. The headphones will be removed at the recovery unit, when patients are fully awake. The music interventions after surgery are each 30 minutes long and takes place twice a day, in the morning and evening. In each hospital, the best times to start the intervention will be established to assure blinding of both the anaesthesiologists and pain specialists. Subjects in the control arm rest for 30 minutes prior to surgery and the administration of premedication, and wear a headphone without music during surgery. After surgery, they receive regular postoperative care without music interventions. Subjects in the control group are instructed to refrain from much listening to music during the hospital stay. The subjects in both control group and intervention group are requested to self-document all activities performed, such as listening to music, playing video games, using the computer and watching movies and television. Participation ends at the scheduled postoperative check up at the outpatient clinic (see figure 1). All study measurements take place during hospitalization and at the outpatient clinic. No extra visits to the hospital are required.

## Music selection

It has been suggested that individual music preference is important to the effect of a music intervention.(22) Nevertheless, a study has shown that playing music from a preselected playlist by the researcher has the largest beneficial effect on postoperative pain, compared to the subject's own favourite music or preselected music without taking the music preference of the subject into account.(19) However, definite conclusions in this regard cannot be drawn. Furthermore, research in rodents suggests that loud rock music may have a negative effect and may act as a stressor.(23)

Therefore, in collaboration with a specialized music therapist we have composed three music playlists without loud rock music, which the subject can choose from. The playlists are categorized into three different genres of music: pop, lounge, and classical



For peer review only - http://bmjopen.bmj.com/site/about/guidelines.xhtml

music. Subjects can choose from either of these playlists. Subjects may choose a different playlist during surgery. Music will be heard through an on-ear headphone connected to a digital music player. After surgery, subjects in the intervention group may listen to their own preferred music.  Approval from Buma/Stemra, the Dutch collecting society for composers and music publishers, has been received to use any licensed music.

**Anaesthetic treatment**

There is no nationwide standard anaesthesia protocol for the Nuss-procedure in the Netherlands. Therefore anaesthesia protocols differ between centres. Randomization should control for such variation between centres. However it will be analysed statistically.

All centres apply EMLA cream® at the intravenous line insertion site. Furthermore, all patients receive epidural analgesia, which are preferably placed at 5th thoracic level. All centres used long-acting local anaesthetics with an adjuvant epidurally. General anaesthesia was induced and maintained by propofol combined with opioids and neuromuscular relaxation induced by rocuronium. Postoperative analgesia was maintained with a continuous epidural infusion of a long-acting local aesthetic (ropivacaine 0.2% or bupivacaine 0.125%) with an adjuvant. All patients received weight-based doses of paracetamol and an NSAID postoperatively. After epidural removal pain was treated by oral opioids as required.

However, there are some major differences between hospitals: The Emma Children's hospital gives standard premedication with clonidine 150 microgr and 300 mg gabapentine, while the other two hospitals do not give any pharmacological premedication. Furthermore, in Emma Children's hospital patients receive gabapentin 300 mg twice daily for 5 days and receive patient-controlled analgesia with morphine in addition to the epidural catheters. Finally, while Juliana and Sophia Children's hospitals use sufentanil 0.5 microgr/ml as an epidural adjuvant, Emma Children's hospital uses clonidine 1 microgr/ml.

**Outcome parameters**

The primary outcome parameter is pain, defined as the average pain score, as measured by a Visual Analogue Scale (VAS-pain), that patients will report at the third day postoperatively. The scale of the VAS-pain varies from 0 to 100, whereas 0 is defined as



For peer review only - http://bmjopen.bmj.com/site/about/guidelines.xhtml

no pain and 100 as the worst pain imaginable. This scale has been recommended and validated for the measurement of acute pain in children 8 years of age and above and is also sensitive to changes in pain levels postoperatively. (24-26)

Secondary outcome parameters include:

- The morphine consumption in the first three days postoperative as calculated by the Morphine Equivalent Dose Daily/ kilogram (MEDD/kg) and the consumption of other analgesics in milligrams.
- Physiological variables such as heart rate, blood pressure and respiratory rate will be measured throughout their hospital stay.
- Levels of anxiety and distress will be measured before surgery through the State-Trait-Anxiety Inventory for children. This questionnaire consists of two separate, 20-item, self-report rating scales for measuring trait and state anxiety. The trait anxiety is a relatively stable personality disposition, while state anxiety is the situation-related anxiety and this may differ depending on the stress of a particular moment. (27) The questionnaire has been translated into Dutch and has been validated. (28)
- Quality of life will be measured before surgery and at their first check-up at the outpatient clinic through the Child Health Utility Questionnaire (CHU9D). This validated questionnaire consists of 9 items that assess the child's functioning across domains of worry, sadness, pain, tiredness, annoyance, schoolwork/homework, sleep, problems with daily routine, and ability to join in activities. (29-31)
- Postoperative complications and length of hospital stay are recorded.
- The subject's post-procedural pain after three months will be evaluated with the VAS, the CHU9D and the 'TNO questionnaire for sport and physical activity'. This validated Dutch questionnaire assessed a person's daily activities. (32) This questionnaire serves to measure rehabilitation as a derivative of the post-procedural pain. Baseline measurements for the 'TNO questionnaire for sport and physical activity' will also be performed before surgery.
- Considering the potential influence of pain and the use of analgesics on length of stay, cost-effectiveness of the intervention will be determined through a cost-utility analysis.



For peer review only - http://bmjopen.bmj.com/site/about/guidelines.xhtml

**Eligibility criteria**

Potential subjects visiting the outpatient clinic of the three paediatric surgery departments involved will be informed about our study. A member of the research team undertakes the initial screening for eligibility. The following inclusion criteria apply:

- Age 12 – 18 years
- Scheduled for primary PE repair according to the Nuss-procedure with either one or multiple bars
- Postoperatively, initial placement of a thoracic epidural or both thoracic epidural and patient-controlled analgesia system
- Good knowledge of the Dutch language, by both patients and parents
- Written informed consent. Additional written informed consent by parents or legal guardian is only necessary for children under the age of 16 years.

The following exclusion criteria apply:

- Hearing impairment
- Secondary PE surgery or other prior thoracic surgery
- Known severe mental or psychiatric disorder
- Known impaired communication with patient and parents as collected
- Presence of chronic pain syndrome: ongoing pain lasting longer than 3 months or ongoing pain lasting longer than the reasonably expected healing time for the involved tissues)

One week after being informed about the study, eligible subjects will be called by telephone to inquire if they wish to participate.

**Sample size**

A power calculation was performed by department of Biostatistics of the Erasmus Medical Centre for the primary outcome parameter: pain, defined as the average pain score, as measured by a Visual Analogue Scale (VAS), that patients will report at the third day postoperatively. Evidence on the effects of recorded music interventions prior, during and after surgery in PE repair is lacking. However, a recent meta-analysis, which investigated music interventions on pain in surgical patients, found an overall effect size measured as the Cohen's delta of -0.50 (CI -0.66;-0.34).(19)



For peer review only - http://bmjopen.bmj.com/site/about/guidelines.xhtml

We assumed a low correlation between the VAS score preoperatively and postoperatively of 0.3. Thus, to obtain a power of 90% using a two-sided significance level of $p<0.05$, each study arm requires 77 subjects. To account for dropouts, we will include 85 subjects per study arm, resulting in a total sample size of 170.

## Statistical analysis

The main study endpoint will be the VAS-pain score reported by the subject during the length of hospital stay, three times a day. The mean VAS-pain score of each day will be calculated per subject. The mean VAS-pain scores between the music and control group on the third day will be compared with an ANCOVA test, with adjustment for the effects of centre and baseline VAS-pain score. The main analysis will be based on the intention-to-treat principle. In case of non-compliance, a sensitivity analysis will be performed using per-protocol analyses. A two sided p-value of <0.05 will be considered to be statistically significant. For the primary outcome parameter, only the available data will be analysed (no imputation of missing data).

In a sensitivity analysis, we will also adjust for possible confounder variables in the linear regression model for the following variables: age, gender, Body Mass Index, and epidural use. Finally, we will also perform a second sensitivity analysis to determine if the effectiveness of the intervention depends on the type of music chosen, by adding these genres as categories to the linear regression model.

The VAS score of each time point will be analysed using a linear mixed model, with the baseline value (observed before surgery), group (control arm or intervention arm), centre, and time point, and the interaction between group and time point as independent variables. Total consumption of analgesics and type of analgesics in milligrams will be added to the analyses. Also an interaction effect of centre and group will be examined, due to variation in anaesthesia protocols in the participating centres. Using information criteria, it will be determined if it is necessary to add a random intercept and/or random slope of time point to this model, to account for the within-subject correlations. If required, a transformation of the outcome will be applied to ensure normality of the model residuals.

The secondary outcome parameters will be analysed as follows.



For peer review only - http://bmjopen.bmj.com/site/about/guidelines.xhtml

• Morphine equivalence daily dose/kilogram (MEDD/kg) and total dosage of other analgesia:

There may be differences between centres in usage of patient controlled analgesia and epidural anaesthesia. Therefore, the difference between the intervention group and the control group will be tested using multiple linear regression, with adjustment for the effects of centre. When necessary, an appropriate transformation of the outcome (MEDD/kg) or total dosage of other analgesia in milligrams will be performed to achieve a normal distribution of the residuals.

• Score on State-Trait-Anxiety-Inventory questionnaire (STAI) and Health related quality of life (HRQoL):

The scores of the STAI and HRQoL questionnaires will be compared between groups using analysis of covariance, with group, centre, and the baseline STAI score and HRQoL score before the intervention or the resting period as independent variables.

• Physiologic measurements, including blood pressure, heart rate and respiratory rate:

These variables will be analysed using a linear mixed model, with the baseline value (observed before surgery), group (control arm or intervention arm), centre, time point, and the interaction between group and time point as independent variables.

Using information criteria, it will be determined if it is necessary to add to a random intercept and/or random slope of time point to this model, to account for the within subject correlations. If necessary, a transformation of the outcome will be applied to ensure normality of the model residuals.

• Complications, like post-operative ileus (n of days), nausea/vomiting (*n* of days and also anti-emetics used), pruritus:

The duration of post-operative ileus, nausea, and vomiting will be compared between groups using a Mann-Whitney test, stratified by centre (i.e. a Van Elteren test). The percentage of patients with pruritus will be compared between groups using a stratified chi-square test.

• Length of hospital stay (n of days):

The length of hospital stay will be compared between groups using a Mann-Whitney test, stratified by centre (i.e. a Van Elteren test).

**Economic evaluation**



For peer review only

We will analyse the cost-effectiveness of the music intervention versus 'standard care' from a health care perspective, using the techniques of cost-effectiveness analysis and cost-utility analysis and following recommended methods for economic evaluations. (33)

Medical costs (i.e., costs within the health care sector) will be analysed, including costs of surgeries, hospital days (on the ward or ICU), medications (such as analgesics), diagnostic radiography, and intercollegiate consultations. For the intervention group, costs of the music intervention will be added, mainly consisting of a Spotify™ subscription. In addition, costs of health care use after the initial hospitalization will be calculated (e.g., outpatient visits, consultations by telephone, (pain) medication, and rehabilitation). Resource consumption for all these items will be derived from electronic databases at the participating centres and from a questionnaire (based on the iMTA Medical Consumption Questionnaire). (34) Unit prices (calculated using economic cost prices or standard prices) will be multiplied by the quantities for each resource used, and then summed over the separate types of resource to give a total cost per patient. Non-medical costs (e.g., out-of-pocket costs and costs of productivity losses incurred by the parents) will be ignored in this study, as these are expected to be relatively minor and not to differ notably between the study groups.

Regarding the patient outcomes, the economic evaluation will look at pain (as measured by the VAS) and HRQoL measured by the CHU9D. The CHU9D is a preference-based measure of HRQoL allowing for the calculation of QALYs, which is a commonly used health outcome measure to calculate the benefits of new interventions within cost-utility analyses for economic evaluation. QALYs will be calculated based on the CHU9D and using linear interpolation between measurement points.

Building on these data on costs and patient outcomes, incremental cost-effectiveness ratios (ICERs) will be calculated, expressed as incremental costs to obtain a reduction of 1 additional unit (10 mm or 1 cm) in the VAS score and as incremental costs per QALY gained. Otherwise, the economic evaluation will focus on dominance of one treatment over the other with respect to lower cost and greater effect. The time horizon of the analysis will be the 3-months follow-up period (starting at the beginning of the hospital



For peer review only - http://bmjopen.bmj.com/site/about/guidelines.xhtml

admission for the PE repair). As a consequence, discounting will not be necessary. Analysis of uncertainty is illustrated through cost-effectiveness planes (via bootstrapping). Sensitivity analysis will be performed to assess the robustness of the analysis to certain assumptions.

## Trial monitoring

An independent trial monitor overseeing all aspects of design, delivery and quality assurance has been appointed by the sponsor, the head of the department of paediatric surgery of the Erasmus Medical Centre. The trial will be monitored at least once per year and a written monitor report will be submitted to the sponsor after each trial-site visit or trial-related communication.

## Data management

Participant data are stored on a secure database in accordance with the General Data Protection Regulations (2018). Data are handled confidentially, de-identified and coded with a unique study number. Published data from this study cannot be traced to a specific subject. Data management for the study was done through OpenClinica and LimeSurvey. Study staff assigned to manage data has access to the OpenClinica and LimeSurvey application and is required to login via an individualized username and password combination. Study staff located at other institutions only has access to the data collected at their sites. The local investigators will safeguard the key that links the unique study number to the patients name at a separate server.

Trial documentation and data will be archived for at least 10 years after completion of the trial.

## Patient and public involvement statement

Patients undergoing PE repair prior to the start of this study evaluated and helped us composing our preselected music playlists.



For peer review only - http://bmjopen.bmj.com/site/about/guidelines.xhtml

## *Ethics and dissemination*

### Ethics

This study protocol has been reviewed and approved by the medical ethics review board at the Erasmus Medical Centre, in Rotterdam on 5 September 2018. This study is being conducted according to the principles of the Declaration of Helsinki (64$^{th}$ WMA General Assembly, Fortaleza, Brazil, October 2013) and in accordance with the Medical Research Involving Human Subject Act (Dutch: WMO). The trial is registered with the Netherlands Trial Register NL6863. To prohibit playing music on the operating room and testing the epidural sensory block daily were approved and implemented as a minor amendment on 9 October 2019.

### Benefits and risks assessment, group relatedness

There are no risks associated with listening to music, except potential hearing damage. To prevent hearing damage, the music administered on the headphones will be adjusted to a maximum of 60 dB, which is the advised loudness of a music intervention in medical care.(35) The maximum dB advised to be exposed to for forty hours a week is 80 dB. (36) Therefore, risk of participation can be considered negligible and the burden minimal. During the informed consent process, it will be made clear that participation in this study has no direct benefits to the patient, and that refusal to participate will not have impact on the care received by any of the medical staff. PE is preferably corrected at age 12-18. This study therefore cannot be conducted without the participation of this group.

All adverse events will be documented. Music intervention itself however, is considered harmless and safe. Therefore, we expect no intervention-related serious adverse events or any other disadvantages for participants in this study.

### Dissemination

The research team is committed to full disclosure of the results of the trial. Findings will be reported in accordance with CONSORT guidelines and we aim to publish in high impact journals. Given the multitude of outcome parameters, results will be divided over several papers. The funder will take no role in the analysis or interpretation of trial results.



For peer review only - http://bmjopen.bmj.com/site/about/guidelines.xhtml

**Authors' contribution:** Each author has contributed significantly to, and is willing to take public responsibility for, one or more aspects of the study. JF Jeekel and RMH Wijnen conceived the study idea. RJ Billar coordinated the research protocol and wrote the first draft of the manuscript. RJ Billar, AYR, Kühlmann, JM Schnater, J Vlot, JJP Tomas, GW Zijp, M Rad, SA de Beer, MF Stevens, MJ Poley, J van Rosmalen, JF Jeekel, and RMH Wijnen critically revised the manuscript. All authors have seen and approved the final version of the manuscript being submitted. The article is the authors' original work, has not received prior publication and is not under consideration for publication elsewhere.

**Conflicts of interest:** none declared.

**Funding:** This research is funded by the Erasmus Medical Centre, Rotterdam, the Netherlands.

**Patient consent:** Not required

**Ethical approval:** The study protocol has received ethical approval by the medical ethical review committee at the Erasmus Medical Centre, in Rotterdam prior to the beginning of the study.

For peer review only



For peer review only - http://bmjopen.bmj.com/site/about/guidelines.xhtml

# References

1. Lam MW, Klassen AF, Montgomery CJ, LeBlanc JG, Skarsgard ED. Quality-of-life outcomes after surgical correction of pectus excavatum: a comparison of the Ravitch and Nuss procedures. J Pediatr Surg. 2008;43(5):819-25.
2. Kelly RE, Jr., Cash TF, Shamberger RC, Mitchell KK, Mellins RB, Lawson ML, et al. Surgical repair of pectus excavatum markedly improves body image and perceived ability for physical activity: multicenter study. Pediatrics. 2008;122(6):1218-22.
3. Frantz FW. Indications and guidelines for pectus excavatum repair. Curr Opin Pediatr. 2011;23(4):486-91.
4. Fonkalsrud EW. Current management of pectus excavatum. World J Surg. 2003;27(5):502-8.
5. Mao YZ, Tang S, Li S. Comparison of the Nuss versus Ravitch procedure for pectus excavatum repair: an updated meta-analysis. J Pediatr Surg. 2017;52(10):1545-52.
6. Abid I, Ewais MM, Marranca J, Jaroszewski DE. Pectus Excavatum: A Review of Diagnosis and Current Treatment Options. J Am Osteopath Assoc. 2017;117(2):106-13.
7. Dean C, Etienne D, Hindson D, Matusz P, Tubbs RS, Loukas M. Pectus excavatum (funnel chest): a historical and current prospective. Surg Radiol Anat. 2012;34(7):573-9.
8. Fonkalsrud EW, Beanes S, Hebra A, Adamson W, Tagge E. Comparison of minimally invasive and modified Ravitch pectus excavatum repair. J Pediatr Surg. 2002;37(3):413-7.
9. Molik KA, Engum SA, Rescorla FJ, West KW, Scherer LR, Grosfeld JL. Pectus excavatum repair: experience with standard and minimal invasive techniques. J Pediatr Surg. 2001;36(2):324-8.
10. Papic JC, Finnell SM, Howenstein AM, Breckler F, Leys CM. Postoperative opioid analgesic use after Nuss versus Ravitch pectus excavatum repair. J Pediatr Surg. 2014;49(6):919-23.
11. St Peter SD, Weesner KA, Weissend EE, Sharp SW, Valusek PA, Sharp RJ, et al. Epidural vs patient-controlled analgesia for postoperative pain after pectus excavatum repair: a prospective, randomized trial. J Pediatr Surg. 2012;47(1):148-53.
12. McCaffrey R, Frock TL, Garguilo H. Understanding chronic pain and the mind-body connection. Holist Nurs Pract. 2003;17(6):281-7; quiz 8-9.
13. Good M. Effects of relaxation and music on postoperative pain: a review. J Adv Nurs. 1996;24(5):905-14.
14. Voss JA, Good M, Yates B, Baun MM, Thompson A, Hertzog M. Sedative music reduces anxiety and pain during chair rest after open-heart surgery. Pain. 2004;112(1-2):197-203.
15. Nguyen TN, Nilsson S, Hellström AL, Bengtson A. Music therapy to reduce pain and anxiety in children with cancer undergoing lumbar puncture: A randomized clinical trial. J Pediatr Oncol Nurs. 2010;27(3):146-55.
16. Nilsson U, Rawal N, Enqvist B, Unosson M. Analgesia following music and therapeutic suggestions in the PACU in ambulatory surgery; A randomized controlled trial. Acta Anaesthesiol Scand. 2003;47(3):278-83.
17. Wang Y, Dong Y, Li Y. Perioperative psychological and music interventions in elderly patients undergoing spinal anesthesia: Effect on anxiety, heart rate variability, and postoperative pain. Yonsei Med J. 2014;55(4):1101-5.
18. Klassen JA, Liang Y, Tjosvold L, Klassen TP, Hartling L. Music for pain and anxiety in children undergoing medical procedures: a systematic review of randomized controlled trials. Ambul Pediatr. 2008;8(2):117-28.



For peer review only - http://bmjopen.bmj.com/site/about/guidelines.xhtml

19. Kuhlmann AYR, de Rooij A, Kroese LF, van Dijk M, Hunink MGM, Jeekel J. Meta-analysis evaluating music interventions for anxiety and pain in surgery. Br J Surg. 2018;105(7):773-83.
20. Fu VX, Oomens P, Klimek M, Verhofstad MHJ, Jeekel J. The Effect of Perioperative Music on Medication Requirement and Hospital Length of Stay: A Meta-analysis. Ann Surg. 2019.
21. van der Heijden MJ, Oliai Araghi S, Jeekel J, Reiss IK, Hunink MG, van Dijk M. Do Hospitalized Premature Infants Benefit from Music Interventions? A Systematic Review of Randomized Controlled Trials. PLoS One. 2016;11(9):e0161848.
22. Kaempf G, Amodei ME. The effect of music on anxiety. A research study. AORN J. 1989;50(1):112-8.
23. Kuhlmann A. Music seems to affect rodents: A systematic review of experimental research. 2017.
24. McGrath PJ, Walco GA, Turk DC, Dworkin RH, Brown MT, Davidson K, et al. Core outcome domains and measures for pediatric acute and chronic/recurrent pain clinical trials: PedIMMPACT recommendations. J Pain. 2008;9(9):771-83.
25. Huguet A, Stinson JN, McGrath PJ. Measurement of self-reported pain intensity in children and adolescents. J Psychosom Res. 2010;68(4):329-36.
26. Tyler DC, Tu A, Douthit J, Chapman CR. Toward validation of pain measurement tools for children: a pilot study. Pain. 1993;52(3):301-9.
27. Moerman N, van Dam FS, Muller MJ, Oosting H. The Amsterdam Preoperative Anxiety and Information Scale (APAIS). Anesth Analg. 1996;82(3):445-51.
28. Ploeg HMvd DP, Spielberger CD, Defares PB, Spielberger CD. Handleiding bij de Zelf-Beoordelings Vragenlijst ZBV : een nederlandstalige bewerking van de Spielberger State- Trait Anxiety Inventory STAI-DY. Lisse: Swets & Zeitlinger. 1980.
29. Stevens K. Valuation of the Child Health Utility 9D Index. Pharmacoeconomics. 2012;30(8):729-47.
30. Stevens K, Ratcliffe J. Measuring and valuing health benefits for economic evaluation in adolescence: an assessment of the practicality and validity of the child health utility 9D in the Australian adolescent population. Value Health. 2012;15(8):1092-9.
31. Ratcliffe J, Stevens K, Flynn T, Brazier J, Sawyer M. An assessment of the construct validity of the CHU9D in the Australian adolescent general population. Qual Life Res. 2012;21(4):717-25.
32. Ooijendijk. Advies Consensus Vragenlijsten Sport en Bewegen. 2007.
33. National Health Care Institute. Guidelines for economic evaluations of health care [in Dutch]. National Health Care Institute, Diemen. 2015.
34. Bouwmans C H-vRL, Koopmanschap M, Krol M, Severens H, Brouwer W iMTA Medical Consumption Questionnaire. Institute for Medical Technology Assessment. Erasmus Universiteit Rotterdam, Rotterdam. 2013.
35. Nilsson U. The anxiety- and pain-reducing effects of music interventions: a systematic review. AORN J. 2008;87(4):780-807.
36. Marinus E, Alphen WJTv. Geluid en trillingen. Zeist: Kerckebosch Uitgeverij|Studiecentrum; 2013.



For peer review only - http://bmjopen.bmj.com/site/about/guidelines.xhtml

## Figure legends

Figure 1 Flow chart of study interventions and assessments

For peer review only



For peer review only - http://bmjopen.bmj.com/site/about/guidelines.xhtml

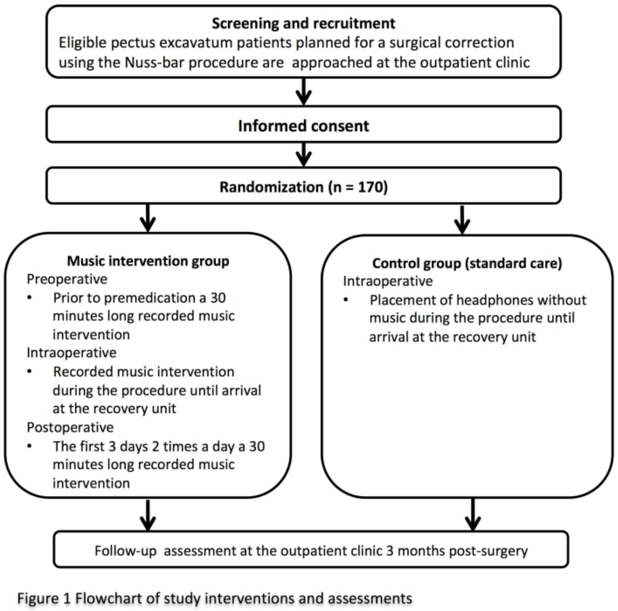


Figure 1 Flow chart of study interventions and assessments

STANDARD PROTOCOL ITEMS: RECOMMENDATIONS FOR INTERVENTIONAL TRIALS

SPIRIT 2013 Checklist for the IMPECT trial (Interventions with Music in Pectus Excavatum Treatment): Recommended items to address in a clinical trial protocol and related documents*

| Section/item | Item No | Description | Addressed on page number in original protocol |
|---|---|---|---|
| **Administrative information** | | | |
| Title | 1 | Descriptive title identifying the study design, population, interventions, and, if applicable, trial acronym | 1 |
| Trial registration | 2a | Trial identifier and registry name. If not yet registered, name of intended registry | 41 |
| | 2b | All items from the World Health Organization Trial Registration Data Set | Yes, www.trialregister.nl |
| Protocol version | 3 | Date and version identifier | 1 |
| Funding | 4 | Sources and types of financial, material, and other support | 5 |
| Roles and responsibilities | 5a | Names, affiliations, and roles of protocol contributors | 2, 3, 4 |
| | 5b | Name and contact information for the trial sponsor | 5 |
| | 5c | Role of study sponsor and funders, if any, in study design; collection, management, analysis, and interpretation of data; writing of the report; and the decision to submit the report for publication, including whether they will have ultimate authority over any of these activities | 33, 40, 41 |

For peer review only

| | | | |
|---|---|---|---|
| | 5d | Composition, roles, and responsibilities of the coordinating centre, steering committee, endpoint adjudication committee, data management team, and other individuals or groups overseeing the trial, if applicable (see Item 21a for data monitoring committee) | 40 |
| **Introduction** | | | |
| Background and rationale | 6a | Description of research question and justification for undertaking the trial, including summary of relevant studies (published and unpublished) examining benefits and harms for each intervention | 12, 13, 14 |
| | 6b | Explanation for choice of comparators | 13, 15, 18 |
| Objectives | 7 | Specific objectives or hypotheses | 14 |
| Trial design | 8 | Description of trial design including type of trial (eg, parallel group, crossover, factorial, single group), allocation ratio, and framework (eg, superiority, equivalence, noninferiority, exploratory) | 15 |
| **Methods: Participants, interventions, and outcomes** | | | |
| Study setting | 9 | Description of study settings (eg, community clinic, academic hospital) and list of countries where data will be collected. Reference to where list of study sites can be obtained | 15, 18, 19 |
| Eligibility criteria | 10 | Inclusion and exclusion criteria for participants. If applicable, eligibility criteria for study centres and individuals who will perform the interventions (eg, surgeons, psychotherapists) | 16, 17 |
| Interventions | 11a | Interventions for each group with sufficient detail to allow replication, including how and when they will be administered | 18, 19 |
| | 11b | Criteria for discontinuing or modifying allocated interventions for a given trial participant (eg, drug dose change in response to harms, participant request, or improving/worsening disease) | 30, 31 |
| | 11c | Strategies to improve adherence to intervention protocols, and any procedures for monitoring adherence (eg, drug tablet return, laboratory tests) | 18, 19 |
| | 11d | Relevant concomitant care and interventions that are permitted or prohibited during the trial | 18, 19 |



For peer review only - http://bmjopen.bmj.com/site/about/guidelines.xhtml

| | | | |
|---|---|---|---|
| Outcomes | 12 | Primary, secondary, and other outcomes, including the specific measurement variable (eg, systolic blood pressure), analysis metric (eg, change from baseline, final value, time to event), method of aggregation (eg, median, proportion), and time point for each outcome. Explanation of the clinical relevance of chosen efficacy and harm outcomes is strongly recommended | 23, 24, 25, 26, 27, 28, 29, 30 and Table 1 |
| Participant timeline | 13 | Time schedule of enrolment, interventions (including any run-ins and washouts), assessments, and visits for participants. A schematic diagram is highly recommended (see Figure) | 24, 25, 26, 27, 28, 29, 30, Table 1 |
| Sample size | 14 | Estimated number of participants needed to achieve study objectives and how it was determined, including clinical and statistical assumptions supporting any sample size calculations | 17 |
| Recruitment | 15 | Strategies for achieving adequate participant enrolment to reach target sample size | 18 |
| **Methods: Assignment of interventions (for controlled trials)** | | | |
| Allocation: | | | |
| Sequence generation | 16a | Method of generating the allocation sequence (eg, computer-generated random numbers), and list of any factors for stratification. To reduce predictability of a random sequence, details of any planned restriction (eg, blocking) should be provided in a separate document that is unavailable to those who enrol participants or assign interventions | 24 |
| Allocation concealment mechanism | 16b | Mechanism of implementing the allocation sequence (eg, central telephone; sequentially numbered, opaque, sealed envelopes), describing any steps to conceal the sequence until interventions are assigned | 24 |
| Implementation | 16c | Who will generate the allocation sequence, who will enrol participants, and who will assign participants to interventions | 24 |
| Blinding (masking) | 17a | Who will be blinded after assignment to interventions (eg, trial participants, care providers, outcome assessors, data analysts), and how | 19, 24 |
| | 17b | If blinded, circumstances under which unblinding is permissible, and procedure for revealing a participant's allocated intervention during the trial | NA |
| **Methods: Data collection, management, and analysis** | | | |

| | | | |
|---|---|---|---|
| Data collection methods | 18a | Plans for assessment and collection of outcome, baseline, and other trial data, including any related processes to promote data quality (eg, duplicate measurements, training of assessors) and a description of study instruments (eg, questionnaires, laboratory tests) along with their reliability and validity, if known. Reference to where data collection forms can be found, if not in the protocol | 18, 19, 24, 25, 26, 27, 28, 29, 30 and Table 1 |
| | 18b | Plans to promote participant retention and complete follow-up, including list of any outcome data to be collected for participants who discontinue or deviate from intervention protocols | 19, 30, 31 |
| Data management | 19 | Plans for data entry, coding, security, and storage, including any related processes to promote data quality (eg, double data entry; range checks for data values). Reference to where details of data management procedures can be found, if not in the protocol | 40 |
| Statistical methods | 20a | Statistical methods for analysing primary and secondary outcomes. Reference to where other details of the statistical analysis plan can be found, if not in the protocol | 34, 35, 36, 37 |
| | 20b | Methods for any additional analyses (eg, subgroup and adjusted analyses) | 34, 35, 36 |
| | 20c | Definition of analysis population relating to protocol non-adherence (eg, as randomised analysis), and any statistical methods to handle missing data (eg, multiple imputation) | 15 |
| **Methods: Monitoring** | | | |
| Data monitoring | 21a | Composition of data monitoring committee (DMC); summary of its role and reporting structure; statement of whether it is independent from the sponsor and competing interests; and reference to where further details about its charter can be found, if not in the protocol. Alternatively, an explanation of why a DMC is not needed | 17, 18 |
| | 21b | Description of any interim analyses and stopping guidelines, including who will have access to these interim results and make the final decision to terminate the trial | NA |
| Harms | 22 | Plans for collecting, assessing, reporting, and managing solicited and spontaneously reported adverse events and other unintended effects of trial interventions or trial conduct | 32, 33, 40 |
| Auditing | 23 | Frequency and procedures for auditing trial conduct, if any, and whether the process will be independent from investigators and the sponsor | 40 |
| **Ethics and dissemination** | | | |

| | | | |
|---|---|---|---|
| Research ethics approval | 24 | Plans for seeking research ethics committee/institutional review board (REC/IRB) approval | Is stated in cover letter to REC as required in the Netherlands |
| Protocol amendments | 25 | Plans for communicating important protocol modifications (eg, changes to eligibility criteria, outcomes, analyses) to relevant parties (eg, investigators, REC/IRBs, trial participants, trial registries, journals, regulators) | 40, 41 |
| Consent or assent | 26a | Who will obtain informed consent or assent from potential trial participants or authorised surrogates, and how (see Item 32) | 15, 38 |
| | 26b | Additional consent provisions for collection and use of participant data and biological specimens in ancillary studies, if applicable | NA |
| Confidentiality | 27 | How personal information about potential and enrolled participants will be collected, shared, and maintained in order to protect confidentiality before, during, and after the trial | 24, 40 |
| Declaration of interests | 28 | Financial and other competing interests for principal investigators for the overall trial and each study site | 39 |
| Access to data | 29 | Statement of who will have access to the final trial dataset, and disclosure of contractual agreements that limit such access for investigators | 40, 41, disclosure of contractual agreements at request |
| Ancillary and post-trial care | 30 | Provisions, if any, for ancillary and post-trial care, and for compensation to those who suffer harm from trial participation | 39 |
| Dissemination policy | 31a | Plans for investigators and sponsor to communicate trial results to participants, healthcare professionals, the public, and other relevant groups (eg, via publication, reporting in results databases, or other data sharing arrangements), including any publication restrictions | 41 |
| | 31b | Authorship eligibility guidelines and any intended use of professional writers | 40, 41, In contractual agreements |


For peer review only - http://bmjopen.bmj.com/site/about/guidelines.xhtml

| | | | |
|---|---|---|---|
| | 31c | Plans, if any, for granting public access to the full protocol, participant-level dataset, and statistical code | NA |
| **Appendices** | | | |
| Informed consent materials | 32 | Model consent form and other related documentation given to participants and authorised surrogates | Available on request |
| Biological specimens | 33 | Plans for collection, laboratory evaluation, and storage of biological specimens for genetic or molecular analysis in the current trial and for future use in ancillary studies, if applicable | NA |

*It is strongly recommended that this checklist be read in conjunction with the SPIRIT 2013 Explanation & Elaboration for important clarification on the items. Amendments to the protocol should be tracked and dated. The SPIRIT checklist is copyrighted by the SPIRIT Group under the Creative Commons "Attribution-NonCommercial-NoDerivs 3.0 Unported" license.

For peer review only

| Data category | Information |
|---|---|
| Primary registry and trial identifying number | Dutch Trial Registry, NL6863 |
| Date of registration in primary registry | 22 February, 2018 |
| Secondary identifying numbers | NTR7041 |
| Source(s) of monetary or material support | Erasmus University Medical Centre |
| Primary sponsor | Erasmus University Medical Centre |
| Secondary sponsor(s) | NA |
| Contact for public queries | RJ Billar, MD, (phone: 0031646794742, e-mail: r.billar@erasmusmc.nl) |
| Contact for scientific queries | RJ Billar, MD, (phone: 0031646794742, e-mail: r.billar@erasmusmc.nl)<br>Erasmus University Medical Centre, the Netherlands |
| Public title | Music interventions in operative treatment of funnel chest |
| Scientific title | Music interventions in pectus excavatum treatment (IMPECT trial) |
| Countries of recruitment | The Netherlands |
| Health condition(s) or problem(s) studied | Pectus Excavatum |
| Intervention(s) | Active comparator: recorded music interventions before, during and the first 3 days after operative repair of pectus excavatum<br>Placebo comparator: No music |
| Key inclusion and exclusion criteria | **Inclusion criteria**<br>▪ Age 12 – 18 years |

| | |
|---|---|
| | ▪ Scheduled for primary pectus excavatum repair<br><br>▪ Surgery according to the Nuss-procedure<br><br>▪ Repair with either one or multiple bars<br><br>▪ General anaesthesia during surgery<br><br>▪ Placement of thoracic epidural or both thoracic epidural and patient controlled analgesia system<br><br>▪ Good knowledge of Dutch or English language by patient and parents<br><br>▪ Written informed consent<br><br>**Exclusion criteria**<br>Hearing impairments<br><br>▪ Secondary pectus excavatum surgery<br><br>▪ Other prior thoracic surgery<br><br>▪ No thoracic epidural<br><br>▪ Severe mental or psychiatric disorder<br><br>▪ Impaired communication with patient and parents<br><br>▪ Missing informed consent<br><br>▪ Presence of chronic pain syndrome (defined as ongoing pain lasting longer than 3 months or ongoing pain lasting longer than the reasonable expected healing time for the involved tissues) |
| Study type | Interventional<br>Allocation: randomized<br>Intervention model: two arms, parallel assignment<br>Masking: single blind (anaesthesiologists, statistician) |



For peer review only - http://bmjopen.bmj.com/site/about/guidelines.xhtml

| | |
|---|---|
| | Purpose: prevention |
| Date of first enrolment | January 2019 |
| Target sample size | 170 |
| Recruitment status | Recruiting |
| Primary outcome(s) | Pain measured by the Visual Analogue scale |
| Key secondary outcomes | Anxiety (measured by the state-trait anxiety inventory), vital parameters, (health related) quality of life, rehabilitation, medication use, complication, patient satisfaction, length of hospital stay, costs |

For peer review only
